# Sepiolite-Supported Manganese Oxide as an Efficient Catalyst for Formaldehyde Oxidation: Performance and Mechanism

**DOI:** 10.3390/molecules29122826

**Published:** 2024-06-13

**Authors:** Dongdong Li, Hongyan Liu, Xiaobao He, Yujie Yao, Haoming Liu, Jun Chen, Bin Deng, Xiaobing Lan

**Affiliations:** Hunan Provincial Key Laboratory of Xiangnan Rare-Precious Metals Compounds Research and Application, School of Chemistry and Environmental Science, Xiangnan University, Chenzhou 423000, China; dongdong_live168@163.com (D.L.); lhyjyjy2024@163.com (H.L.); 15323708027@163.com (X.H.); yaoyujie9975@163.com (Y.Y.); 19573590503@163.com (H.L.); jchen4174@xnu.edu.cn (J.C.); dengbinxnu@163.com (B.D.)

**Keywords:** formaldehyde oxidation, sepiolite, manganese oxide, catalysis, mechanism

## Abstract

The current study involved the preparation of a number of MnO_x_/Sep catalysts using the impregnation (MnO_x_/Sep-I), hydrothermal (MnO_x_/Sep-H), and precipitation (MnO_x_/Sep-P) methods. The MnO_x_/Sep catalysts that were produced were examined for their ability to catalytically oxidize formaldehyde (HCHO). Through the use of several technologies, including N_2_ adsorption–desorption, XRD, FTIR, TEM, H_2_-TPR, O_2_-TPD, CO_2_-TPD, and XPS, the function of MnO_x_ in HCHO elimination was examined. The MnO_x_/Sep-H combination was shown to have superior catalytic activities, outstanding cycle stability, and long-term activity. It was also able to perform complete HCHO conversion at 85 °C with a high GHSV of 6000 mL/(g·h) and 50% humidity. Large specific surface area and pore size, a widely dispersed active component, a high percentage of Mn^3+^ species, and lattice oxygen concentration all suggested a potential reaction route for HCHO oxidation. This research produced a low-cost, highly effective catalyst for HCHO purification in indoor or industrial air environments.

## 1. Introduction

Formaldehyde (HCHO) is a gaseous indoor pollutant and possible human carcinogen that is released by paints, plastic cements, construction and furnishing materials, and other consumer goods [1,2,3,4]. Therefore, HCHO removal technologies have been developed to lessen HCHO pollution indoors. Catalytic oxidation is an economical and environmentally benign method of directly breaking down HCHO into CO_2_ and H_2_O [5,6,7,8]. Noble metal catalysts (Pt, Au, Pd, Ag, and Ir) can fully catalyze HCHO at low or ever room temperature [9,10,11,12,13,14]. However, the practical use of catalysts was limited by the high production costs associated with the usage of precious metals. Many non-noble metal oxide catalysts (such as Ce, Mn, Co, Fe, and Cu) have been produced to date for the complete oxidation of HCHO at low temperature [15,16,17,18,19,20,21]. Among them, manganese-based catalysts with various supports, plentiful valence states, structural flexibility, and functional design have garnered the most interest because of their superior catalytic activity at lower temperatures [6,22,23,24,25,26,27]. But the low catalytic activity remains a barrier that these catalysts urgently need to overcome.

Other techniques include supported-manganese-based catalysts, in which manganese oxides are typically placed on high-specific-surface-area carrier materials, like zeolite [28,29,30], activated carbon (AC) [31,32], and MOF [33]. These carrier materials are primarily used to increase the dispersion degree of the active components and the super capture ability of HCHO and serve as a platform for designing and synthesizing manganese oxides with richer oxygen vacancies or metal defects that can activate surface oxygen species and H_2_O to degrade the captured HCHO into harmless CO_2_ and H_2_O. To the best of our knowledge, we reviewed the pertinent papers and discovered that the natural clay mineral sepiolite, with its large specific surface area, stability, and abundance of hydroxyl species, is a perfect carrier material that can avoid an aggregation of active components and capture ability of HCHO [20,34]. In Hunan province, China, sepiolite (Sep) is a naturally occurring phyllosilicate fibrous clay mineral with large reserves and inexpensive pricing (0.4–0.6 $/kg) [35]. It is composed of continuous tetragonally coordinated silicon layers that reverse their orientation every few tetrahedral units and discontinuous octahedrally coordinated magnesium layers. Sepiolite’s unique structure accounts for its homogeneous pore size, elevated porosity, large surface area, and profusion of silanol groups [36,37,38,39]. Although there have been reports of sepiolite in HCHO degradation, most of them have focused on photocatalysis [40,41,42], with thermocatalysis receiving even fewer reports [20,34]. Our research on HCHO oxidation via the use of sepiolite and silver has shown that the catalysts have unique adsorption characteristics and are capable of fully decomposing HCHO at 52 °C into CO_2_ and H_2_O [43]. However, the usage of silver species carries a price risk for the catalyst.

On this basis, based on the effective catalytic ability of manganese and the unique properties of sepiolite, we synthesized three sepiolite–manganese catalysts by impregnation (MnO_x_/Sep-I), precipitation (MnO_x_/Sep-P), and hydrothermal methods (MnO_x_/Sep-H). We then assessed the catalytic activities of these catalysts for the total degradation of HCHO. We found that at lower temperatures, the MnO_x_/Sep-H catalyst exhibited better catalytic efficacy because of the higher ratio of Mn^3+^/Mn^4+^, larger pore size and abundant surface-active oxygen species. Moreover, the technologies of XRD, FTIR, TPD/TPR, XPS, and N_2_ adsorption–desorption were used to demonstrate the important effect that vacancies play on surface oxygen species and H_2_O activation for HCHO oxidation. The potential mechanism for the HCHO oxidation process was finally put forth.

## 2. Results and Discussion

### 2.1. Structural Characterization

**Figure 1:**
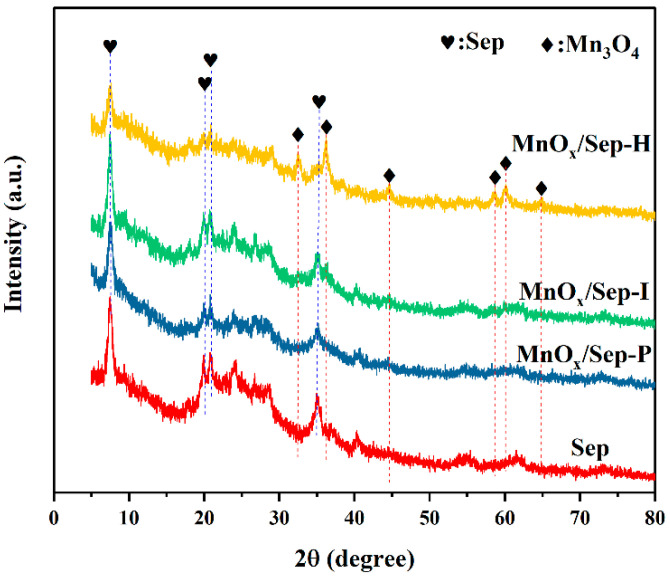
The XRD patterns of Sep, MnO_x_/Sep-P, MnO_x_/Sep-I, and MnO_x_/Sep-H.

The XRD patterns for MnO_x_/Sep-P, MnO_x_/Sep-I, and MnO_x_/Sep-H are shown in Figure 1. The findings showed that the (110), (010), (101), and (010) planes of in Sep were responsible for the diffraction reflections at 2θ = 9.5°, 19.8°, 20.7°, and 32.5 (JCPDS 75-1621) [43,44]. Furthermore, the distinctive diffraction reflections of the Mn_3_O_4_ structure (JCPDS, No. 80-0382) were found at 28.9°, 32.4°, and 36.1°. These peaks might be attributed to the (112), (103), and (211) crystal facets of Mn_3_O_4_, respectively [7,25]. It appears that the structure of Sep remained unaffected by the addition of manganese species. The fact that the Mn_3_O_4_ structure diffraction reflections for MnO_x_/Sep-P and MnO_x_/Sep-I were not readily visible suggests that the Mn_3_O_4_ structures were evenly distributed across the sepiolite surface. However, the Mn_3_O_4_ structure’s diffraction reflections were seen in MnO_x_/Sep-H, indicating that it might produce a Mn_3_O_4_ structure. The existence of Mn_3_O_4_ might promote the formation of Mn-vacancies (Mn_v_) or manganese defects [25,26]. The formation of a Mn_v_ is conducive to the adsorption and activation of oxygen molecules, producing the reactive oxygen species [45,46,47]. Several investigations have demonstrated that the formation of Mn-vacancies or manganese defects in catalysts is advantageous for absorbing oxygen and water molecules, creating surface-active oxygen species, and accelerating the catalytic oxidation of HCHO [17,23,25].

**Figure 2:**
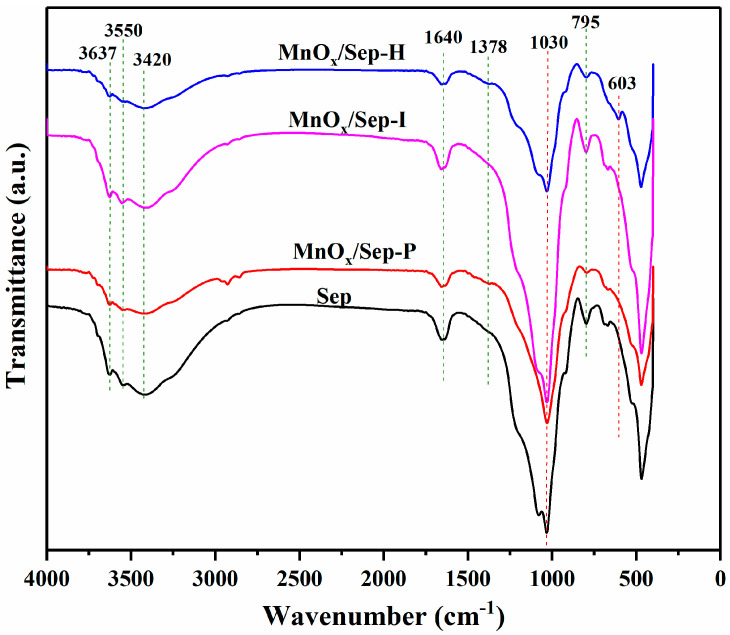
The FTIR spectra of Sep, MnO_x_/Sep-P, MnO_x_/Sep-I, and MnO_x_/Sep-H.

In Figure 2, the catalysts’ FTIR spectra are displayed. For Sep, the -OH groups are represented by the bands near 3420 cm^−1^, the hydroxyl groups in crystalline water and surface adsorption water are represented by the bands at 3637 cm^−1^ and 3637 cm^−1^, and the -OH vibration of H_2_O is responsible for the peaks at 1640 cm^−1^ [36,38,43]. Furthermore, the vibrations of Si-O in the tetrahedral silica sheet are responsible for the peaks at 1030 and 795 cm^−1^ [36,39]. After loading the manganese species, the intensity of vibration of the hydroxyl groups and other group structure did not significantly change for MnO_x_/Sep-P, MnO_x_/Sep-I, and MnO_x_/Sep-H. The results indicated that surface hydroxyl species are abundant, which facilitates HCHO adsorption and may enhance the catalyst’s catalytic activity [48]. Furthermore, for MnO_x_/Sep-H, additional vibration peaks emerged at 603 cm^−1^, which could be related to the vibration of the Mn-O bond [49]. Manganese oxides may have been more firmly bound to Sep as shown by the formation of the Mn-O bond in Mn_3_O_4_, which could have aided in the creation of Mn-vacancies (Mn_v_). This finding was in line with the XRD results as well, which provided more evidence that Mn-vacancies could exist in the MnO_x_/Sep-H catalyst.

**Figure 3:**
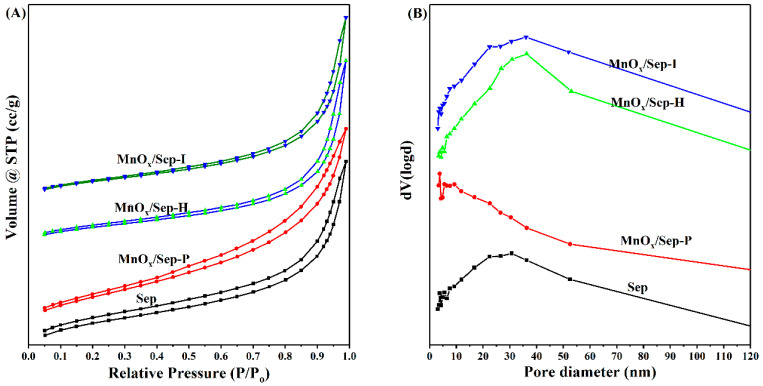
The N_2_ adsorption–desorption curve and pore size distribution patterns of catalysts. (**A**) the N_2_ adsorption–desorption curve; (**B**) the pore size distribution curve.

Based on the previous literature [50,51], the N_2_ adsorption–desorption curve and pore size distribution patterns of Sep, MnO_x_/Sep-P, MnO_x_/Sep-I, and MnO_x_/Sep-H are displayed in Figure 3. Each sample showed a type IV isotherm and a type H4 hysteresis loop, indicating that they were all made of mesoporous material [36,43]. Also, the physical parameters of the mesoporous materials and surface element composition of the samples are displayed in Table 1. The pore diameter was between 10 and 50 nm. The S_BET_, V_pore_, and D_pore_ of Sep were 325.16 m^2^/g, 0.32 cm^3^/g, and 3.82 nm. The partial coverage of the Sep surface pores by Mn species causes the S_BET_ to fall to 229.45, 136.13, and 176.77 m^2^/g after adding manganese species. However, the V_pore_ for the samples were barely altered. In contrast to Sep (3.82 nm), the D_pore_ of MnO_x_/Sep-P and MnO_x_/Sep-I decreased from 3.82 nm to 3.41 nm and 3.42 nm, respectively. But the D_pore_ of MnO_x_/Sep-H (3.82 nm) remained relatively unchanged, indicating that the manganese species did not block the pores of sepiolite in MnO_x_/Sep-H catalysts. HCHO adsorption might be favored by a bigger pore size; hence, it further promotes the catalytic activity of the catalyst.

**Figure 4:**
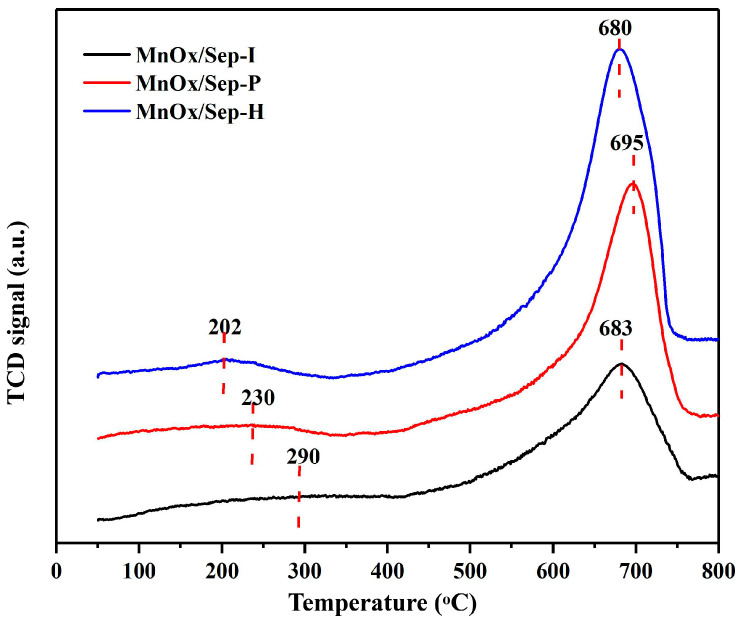
The H_2_-TPR profiles of MnO_x_/Sep-H, MnO_x_/Sep-P, and MnO_x_/Sep-I.

Figure 4 display the H_2_-TPR profiles of the catalysts as prepared. The purpose of the experiment was to assess the reactivity and reducibility of various oxygen species in catalysts. Each sample curve had two distinct reduction peaks, as Figure 4 illustrates. These peaks corresponded to the reduction of bigger-particle manganese oxides (MnO_2_→Mn_2_O_3_→Mn_3_O_4_→MnO) and surface-active oxygen [30,52]. Theoretically, significant reducibility and high reactivity of the oxygen species are indicated by a lower reduction temperature. Since the peak of MnO_x_/Sep-H’s reduction temperature (202 °C) is lower than that of MnO_x_/Sep-P (230 °C) and MnO_x_/Sep-I (290 °C), it may be concluded that MnO_x_/Sep-H’s surface-adsorbed oxygen showed the best reducibility [48]. Furthermore, for MnO_x_/Sep-H, MnO_x_/Sep-P, and MnO_x_/Sep-I, the reduction temperatures were 680 °C, 695 °C, and 683 °C for bigger-particle manganese oxides, and the specific gravity of hydrogen consumption was determined to be 1.8:1.6:1, suggesting that MnO_x_/Sep-H has more lattice oxygen species, which may also promote catalytic activity.

**Figure 5:**
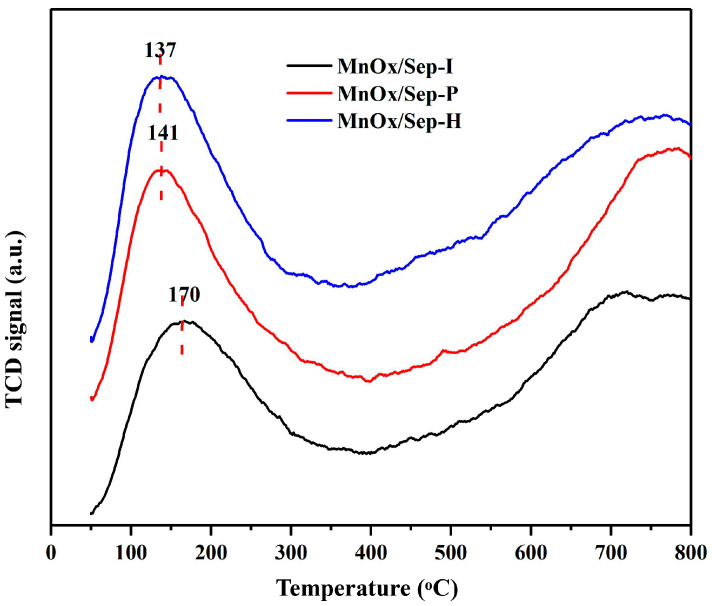
The O_2_-TPD profiles of MnO_x_/Sep-H, MnO_x_/Sep-P, and MnO_x_/Sep-I.

To learn more about the desorption patterns and chemical reactions of various oxygen species, O_2_-TPD was carried out. Two peaks were seen at 100–300 °C and 600–800 °C in the O_2_-TPD profiles of the MnO_x_/Sep-H, MnO_x_/Sep-P, and MnO_x_/Sep-I catalysts in Figure 5. The adsorbed oxygen species (O_2_^−^, O^2−^ or O^−^) at the surface was the first desorption peak and is active for the full oxidation of HCHO. The bulk lattice oxygen (O^2−^) is responsible for other desorption peaks. The desorption temperatures of MnO_x_/Sep-H, MnO_x_/Sep-P, and MnO_x_/Sep-I were 137 °C, 141 °C, and 170 °C, respectively, for surface-adsorbed oxygen species. Furthermore, the specific gravity of oxygen desorption for MnO_x_/Sep-H, MnO_x_/Sep-P, and MnO_x_/Sep-I was determined to be 1.4:1.3:1.2. According to these findings, MnO_x_/Sep-H, MnO_x_/Sep-P, and MnO_x_/Sep-I has a higher concentration of activated oxygen species than the others, which is advantageous for the oxidation of HCHO [48].

**Figure 6:**
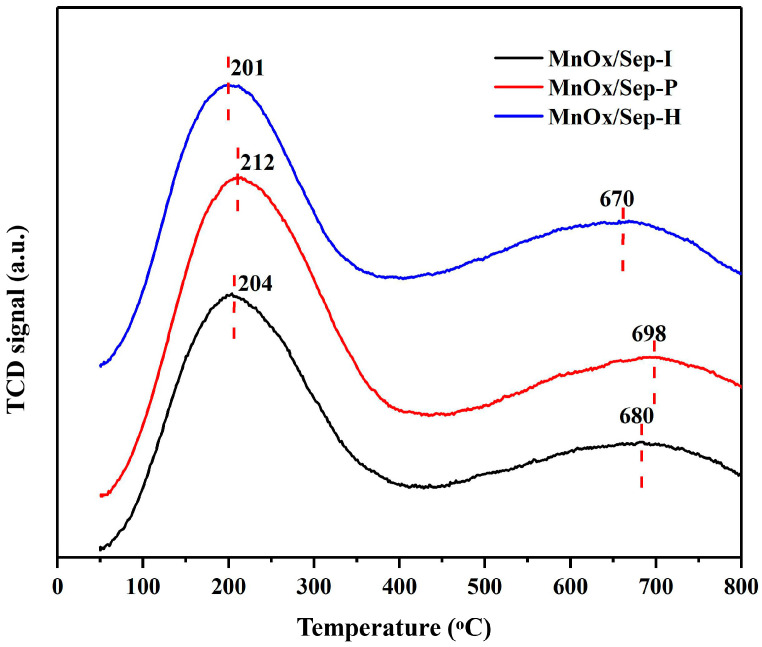
The CO_2_-TPD profiles of MnO_x_/Sep-H, MnO_x_/Sep-P, and MnO_x_/Sep-I.

Furthermore, the basic sites in Figure 6 were identified using CO_2_-TPD profiles. MnO_x_/Sep-H had its first peak at 201 °C, while MnO_x_/Sep-P and MnO_x_/Sep-I had their first peaks at 204 °C and 212 °C, respectively. The reason for this was that compared to the surfaces of MnO_x_/Sep-P and MnO_x_/Sep-I, the basic sites on the MnO_x_/Sep-H surface were weaker. Furthermore, the specific gravity of CO_2_ desorption for MnO_x_/Sep-H, MnO_x_/Sep-P, and MnO_x_/Sep-I was determined to be 1.8:1.7:1.2. According to these findings, MnO_x_/Sep-H was favorable for CO_2_ desorption, which can promote the desorption of CO_2_ products and increase catalytic activity [43]. The CO_2_ desorption peaks of MnO_x_/Sep-H were pushed to lower temperatures in the high-temperature range (500–800 °C) in comparison to those of MnO_x_/Sep-P and MnO_x_/Sep-I. Therefore, the MnO_x_/Sep-H had more basic sites that were advantageous for the adsorption of HCHO on its surface, and its higher catalytic activity was due to its decreased CO_2_ desorption.

**Figure 7:**
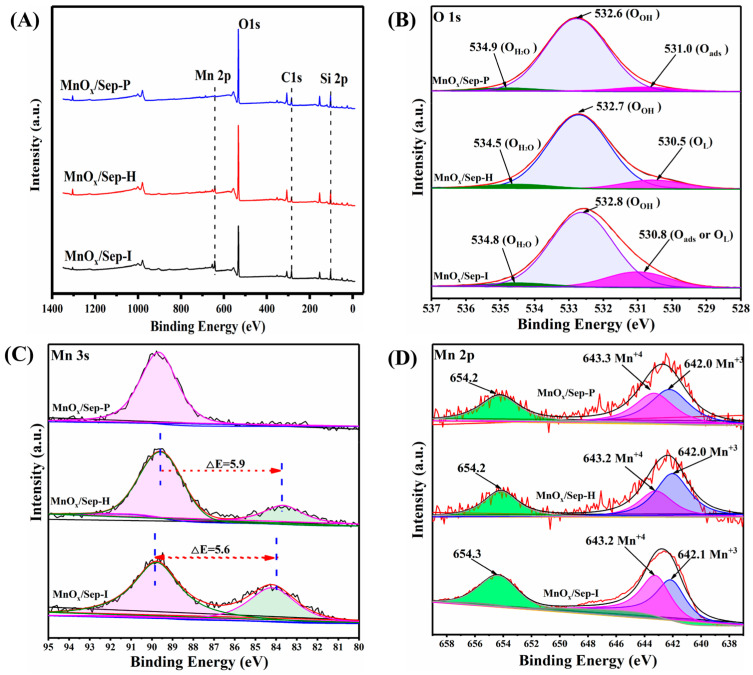
The XPS patterns of wide-scan spectra (**A**), O 1s (**B**), Mn 3s (**C**), and Mn 2p (**D**) of MnO_x_/Sep-H, MnO_x_/Sep-P, and MnO_x_/Sep-I.

The chemical composition of MnO_x_/Sep-H, MnO_x_/Sep-P, and MnO_x_/Sep-I is studied by XPS (Figure 7). In Figure 7A, the wide-scan spectra indicates that Si, O, C, and Mn elements exist in three catalysts. And the C 1s and Si 2p high-resolution XPS spectra are shown in Figures S1 and S2 (Supplementary Materials). Comparing MnO_x_/Sep-P and MnO_x_/Sep-I, the shift of binding energy position appeared in MnO_x_/Sep-H, indicating a stronger interaction between the nanoparticles and the carrier. In addition, the position near 103.9 eV and 103.2 eV correspond to SiO_2_ and Si-O bonds, but the proportion of Si-O bonds in MnO_x_/Sep-H significantly increases, suggesting that there may be a Si-O-metal structure on the surface of MnO_x_/Sep-H, such as Si-O-Mn bonds [38,39]. These visualizations suggest a robust interaction between the active ingredients and the carrier, which is advantageous for the dispersion of active components and the formation of new bonds.

Three different types of oxygen species were found in the core level O 1s spectra (Figure 7B) for MnO_x_/Sep-H, MnO_x_/Sep-P, and MnO_x_/Sep-I. These species include the following: (i) O_L_ or O_ads_ (530.0–530.8 eV); (ii) surface hydroxyl species (O_OH_, 532.6–532.8 eV); and (iii) surface water (O_H2O_, 534.5–534.9 eV) [26,48,53]. Furthermore, according to the XPS-41 software’s fitting results, the proportions of O_L_ or O_ads_ surface O_OH_ and O_H2O_ in MnOx/Sep-P were 6.7%, 89.6%, and 3.8%, were 10%, 85.6%, and 4.3% in MnO_x_/Sep-H, and were 16.4%, 80%, and 3.7% in MnO_x_/Sep-I, respectively. More surface hydroxyl groups were present in three catalytic materials, which was advantageous for the adsorption of HCHO. However, MnO_x_/Sep-I and MnO_x_/Sep-H have greater amounts of O_L_ or O_ads_ than MnO_x_/Sep-P, suggesting that these two catalysts may have more reactive oxygen species and the capacity to activate oxygen, which would aid in the conversion of adsorbed HCHO. On the other hand, MnO_x_/Sep-H exhibits positive-shift binding energies (about 0.5 eV) of the O_L_ or O_ads_ in contrast to MnO_x_/Sep-P and MnO_x_/Sep-I, suggesting the presence of interactions among Sep, O species, and Mn species. In conclusion, the abundance of surface O_OH_ and O_L_ or O_ads_ along with the robust interaction between the Sep, O species, and Mn species all work together to enhance the degradation of HCHO by MnO_x_/Sep-H catalysts.

Figure 7C,D display high-resolution XPS spectra of Mn 3s and Mn 2p. The formula for calculating the average oxidation state (AOS) of the surface Mn in MnO_x_/Sep-H and MnO_x_/Sep-I is AOS = 8.956 − 1.126ΔE, where ΔE is the splitting width of Mn 3s [17,23,54]. For MnO_x_/Sep-H and MnO_x_/Sep-I, the corresponding AOS values were found to be 2.31 and 2.65, respectively. The AOS of the surface Mn for MnO_x_/Sep-I was close to the typical oxidation state of Mn in Mn_3_O_4_ (2.67), whereas MnO_x_/Sep-H catalysts had a lower AOS of surface Mn (2.31), suggesting that it may form the Mn-vacancies or O-vacancies. The Mn 2p3/2 spectra, which were fitted into two peaks (Figure 7C) at around ∼642.1 eV and ∼643.2 eV, which can be attributed to Mn^3+^ and Mn^4+^ species, respectively [21,25,26,48]. According to the XPS-41 software’s fitting results, MnO_x_/Sep-P, MnO_x_/Sep-H, and MnO_x_/Sep-I have Mn^3+^/Mn^4+^ fractions of 1.15:1, 1.67:1, and 1:1, respectively. MnO_x_/Sep-H has a high percentage of Mn^3+^, and the surface Mn^3+^ promotes the development of surface oxygen or Mn-vacancies (O_v_ or Mn_v_), which makes it easier to activate oxygen to surface-active species and improves oxidation capacity. This explains why the MnO_x_/Sep-H catalysts have higher activity in the oxidation of HCHO.

**Figure 8:**
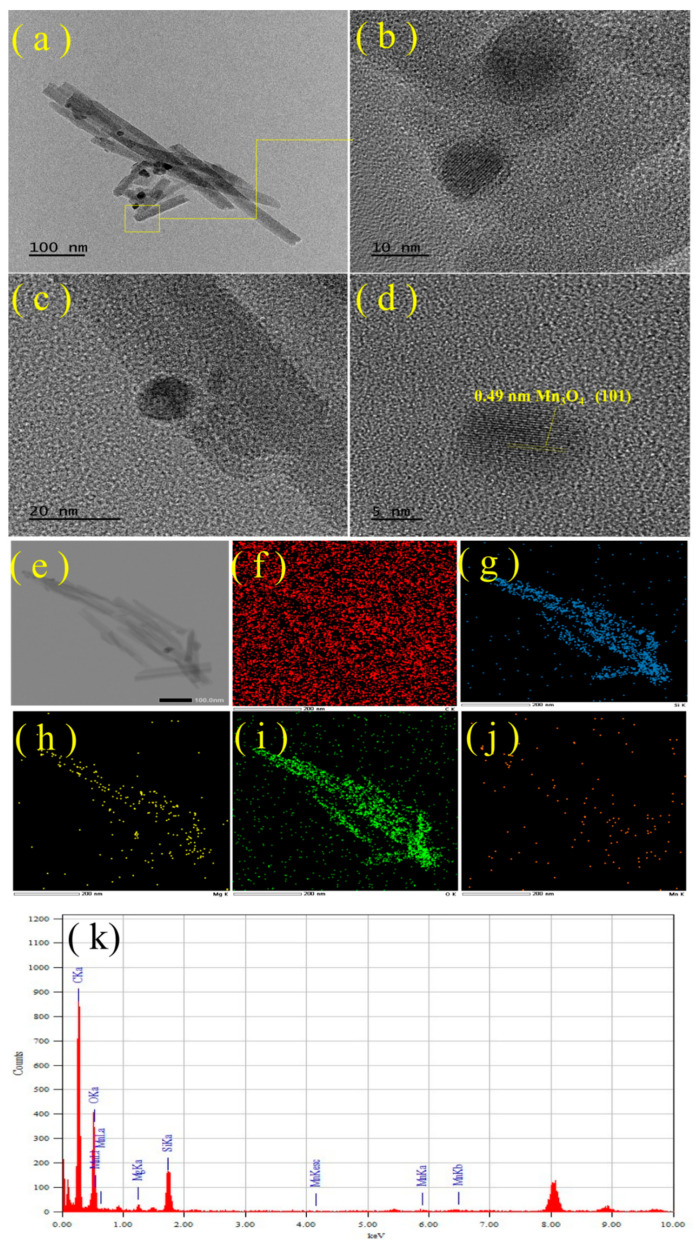
TEM, STEM, and element distribution micrographs of selected samples. (**a**–**d**) TEM micrograph of MnO_x_/Sep-H, (**e**) HAADF-STEM of MnO_x_/Sep-H with element distribution micrographs (**f**–**j**) corresponding to C, Si, Mg, O, and Mn, (**k**) spectra from area of MnO_x_/Sep-H.

The microstructure of the MnO_x_/Sep-H catalyst was obtained through TEM micrographs, as depicted in Figure 8. The sepiolite had a fibrous structure resembling a needle, and manganese nanoparticles were evenly dispersed on the surface of the sepiolite. The average size of these particles was less than 10 nm. Moreover, some of manganese nanoparticles are clearly loaded onto the sepiolite fiber boundaries, which may suggest a strong interaction between surface nanoparticles and sepiolite. The microstructures of various Mn_3_O_4_ samples were observed by TEM. It is evident that micro-conjunctive nanoparticles make up the macroscopic morphology of MnO_x_/Sep-H. The lattice spacing of 0.49 nm observed on MnO_x_/Sep-H is associated with the (101) facets of Mn_3_O_4_, as demonstrated by high-resolution transmission electron microscopy (HRTEM) pictures [25]. Using element distribution micrographs from the HAADF-STEM of MnO_x_/Sep-H, the elements C, Si, Mg, O, and Mn were identified and constructed the catalyst.

### 2.2. Catalytic Performance

**Figure 9:**
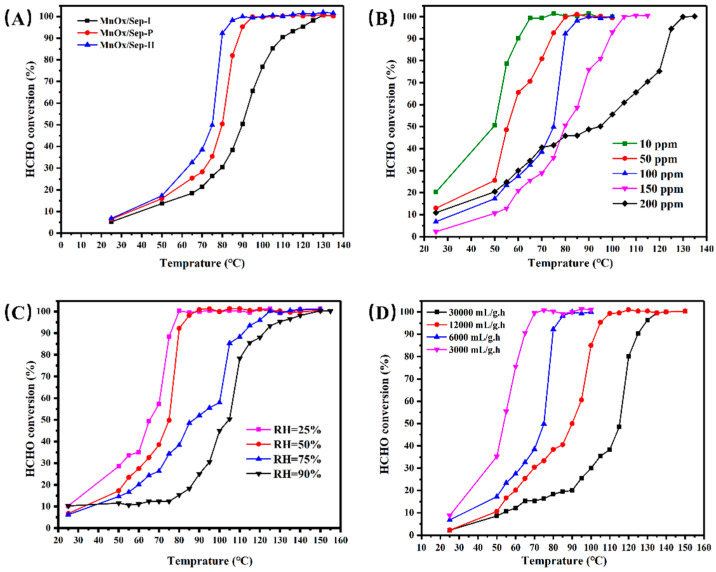
The performance of catalytic oxidation of HCHO. (**A**) The catalytic activity of MnO_x_/Sep-H, MnO_x_/Sep-P, and MnO_x_/Sep-I (reaction conditions: 100 ppm of HCHO, WHSV 6000 mL/(g·h)); (**B**) Effects of different HCHO concentrations on catalytic oxidation of HCHO over MnO_x_/Sep-H (reaction conditions: RH of 50% and GHSV 6000 mL/(g·h)); (**C**) Effects of different reaction humidities on catalytic oxidation of HCHO over MnO_x_/Sep-H (reaction conditions: 100 ppm of HCHO and GHSV 6000 mL/(g·h)); (**D**). Effects of different GHSV on MnO_x_/Sep-H catalyst activity. (reaction conditions: 100 ppm of HCHO and RH of 50%).

Figure 9A displays the HCHO conversion over MnO_x_/Sep-H, MnO_x_/Sep-P, and MnO_x_/Sep-I. These catalysts have the ability to fully oxidize HCHO at varying temperatures, indicating that the manganese active components supported by sepiolite exhibit activity with different methods of preparation. The phenomenon in question could potentially be attributed to the combined action of activating manganese active components and sepiolite’s efficient uptake of HCHO. However, the differences in MnO_x_/Sep-H, MnO_x_/Sep-P, and MnO_x_/Sep-I catalytic performance are clearly visible. With reaction conditions of 100 ppm of HCHO, 50% reaction humidity, and 6,000 mL/(g·h), the MnO_x_/Sep-H catalyst can totally oxidize HCHO to CO_2_ at 85 °C; however, it is required at 95 °C and 125 °C over MnO_x_/Sep-P and MnO_x_/Sep-I with the same reaction circumstances, respectively. MnO_x_/Sep-H clearly demonstrates superior catalytic performance. The Mn_3_O_4_ structure was created by manganese species, as shown by XRD, FTIR, and XPS data. Manganese species have the potential to produce Mn-vacancies (Mnv), which is favorable for the adsorption and activation of oxygen molecules and generates reactive oxygen species (ROS) that enhance catalytic performance. Furthermore, MnO_x_/Sep-H has the biggest pore size and least amount of variation in comparison to sepiolite among the three catalysts, although it has the smallest specific surface area. A larger pore size may be beneficial for capturing and storing HCHO. Additionally, MnO_x_/Sep-H demonstrated superior oxygen activation and product desorption abilities in H_2_-TPR, O_2_-TPD, and CO_2_-TPD, all of which aided in the promotion of HCHO oxidation.

Figure 9B–D illustrate the effects of the HCHO inlet concentration, reaction humidity, and space velocity (GHSV) on HCHO catalytic oxidation over MnO_x_/Sep-H. According to the results, at 65 °C, 80 °C, 85 °C, 105 °C, and 130 °C, with HCHO concentrations of 10, 50, 100, 150, and 200, and under reaction conditions of 6000 mL/(g·h) and 50% reaction humidity, MnO_x_/Sep-H can totally oxidize HCHO. And under reaction conditions of GHSV of 6000 mL/(g·h) and 100 ppm of HCHO, MnO_x_/Sep-H can oxidize HCHO entirely at 75 °C, 85 °C, 105 °C, and 150 °C at reaction humidities of 25%, 50%, 75%, and 90%. Furthermore, the HCHO may be fully oxidized at 50 °C, which exhibits the highest catalytic performance when compared to MnO_x_/Sep-H at the lowest temperatures of 65 °C, 85 °C, 105 °C, and 130 °C under reaction conditions of 50% reaction humidity and 100 ppm HCHO. And the oxidative degradation temperature increased with the increment in GHSV.

**Figure 10:**
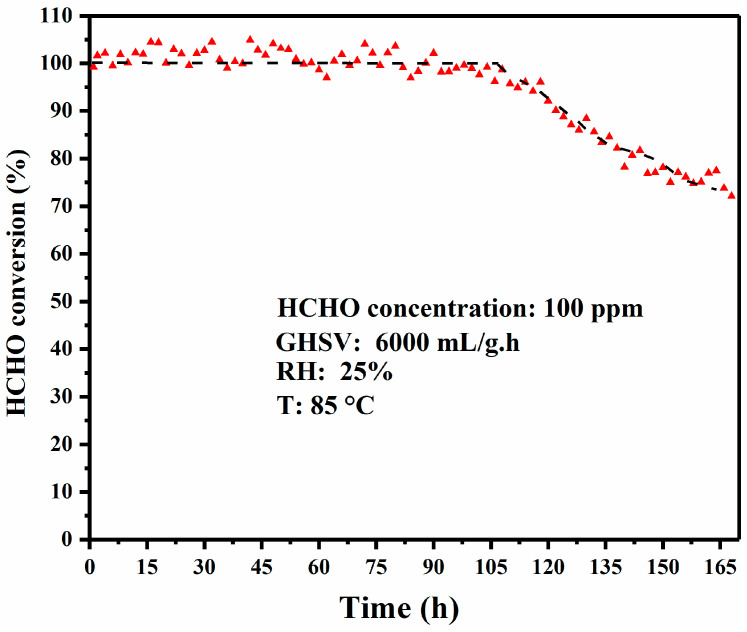
The stability of oxidative degradation of 100 ppm HCHO over MnO_x_/Sep-H at 85 °C, GHSV of 6000 mL/(g·h) and RH of 25%.

Figure 10 demonstrates the stability of oxidative degradation of 100 ppm HCHO over MnO_x_/Sep-H at 85 °C, 25% relative humidity, and 6000 mL/(g·h) GHSV. This indicates that the catalyst sustains the activity even when the reaction period reaches 105 h. Nevertheless, the catalytic activity gradually decreases as the reaction goes on. As the reaction goes on for more than 160 h, the catalytic activity drops to roughly 70%. This could be caused by the active components’ decreased capacity to activate molecular oxygen and the sepiolite’s ability to capture HCHO, as well as the continual accumulation of intermediate and target products that occupy the active sites.

### 2.3. Reaction Mechanism of HCHO Oxidation over MnO_x_/Sep-H

**Figure 11:**
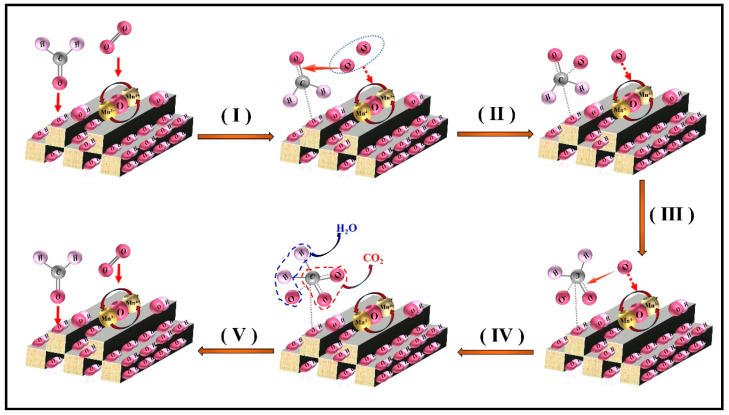
Schematic of proposed mechanism for HCHO removal process over MnO_x_/Sep-H catalyst. HCHO.

Based on the acquired data and earlier studies [43], a potential mechanism of the catalytic oxidation of HCHO on the MnO_x_/Sep-H catalyst is shown in Figure 11. Firstly, it involved the adsorption of HCHO and O_2_ onto the catalyst surface through the hydroxyl groups and d-orbital vacancies of Mn at step **I**, respectively. Secondly, the adsorbed O_2_ split to active oxygen radicals (O^−^) on Mn-vacancies, simultaneously converting Mn^3+^ to Mn^4+^ at step **II**. In this stage, oxygen radicals (O^−^) attacked the C=O bond of adsorbed HCHO, converting it to HCOOH (formate). Thirdly, HCOOH was further oxidized to CO_2_ and H_2_O by another oxygen radical (O^−^) at step **III** and step **IV** which has the ability to attack the C-O bond of formate species. Finally, the Mn^4+^ converts to Mn^3+^, and the occupied hydroxyl groups and d-orbital vacancies of Mn were also released at step **V**, providing exceptional stability for recycling. Therefore, with the exception of physisorption on sepiolite nanofibers and oxidation on MnO_x_ active sites, the catalytic oxidation of HCHO on MnO_x_/Sep-H catalysts is therefore greatly enhanced by the synergistic impact of conversion between Mn^3+^ to Mn^4+^ and hydroxyl groups.

## 3. Materials and Methods

### 3.1. Materials

Sepiolite ore (30% purity) was obtained from Hunan Yuanyuan Sepiolite Technology Co. LTD (Xiangtan, China), and the high-purity sepiolite (Sep) was prepared through our previously reported methods [43]. Potassium permanganate (KMnO_4_) was supplied by Sinopharm Chemical Reagent Co., Ltd. (Beijing, China). L-ascorbic acid was supplied by Kemiou Chemical Reagent Co., Ltd. (Tianjin, China). NaOH was obtained from Xilong Scentific Co., Ltd. (Guangzhou, China).

### 3.2. Catalysts Preparation

#### 3.2.1. Synthesis of MnO_x_/Sepolite-I Nanoparticles by Impregnation Method

The MnO_2_/Sepolite-I was obtained by the impregnation method as follows. First, sepiolite fibers were dispersed in deionized aqueous solution at a mass ratio of 1:40, and the mixture was stirred at room temperature for 24 h. Then, a certain amount of potassium permanganate was dispersed in the aqueous solution and fully dissolved, and then, it was slowly added dropwise to the sepiolite fiber aqueous suspension at a rate of 2 s/drop and stirred under reflux for 6 h at 105 °C. Subsequently, a quantitative amount of L-ascorbic acid was dissolved and slowly added dropwise to the above mixture at a speed of 2 s/drop and stirred for 6 h at 105 °C under reflux. After the reaction, the mixture was centrifuged to retain the solid; then, the solid was washed three times with absolute ethanol and water and dried overnight at 80 °C. Lastly, the dried solid was ground into powder and roasted at 350 °C for 5 h to obtain MnO_2_/Sepolite by the impregnation method. The sample was labeled as MnO_2_/Sepolite-I.

#### 3.2.2. Synthesis of MnO_x_/Sepolite-P Nanoparticles by Precipitation Method

The synthesis of MnO_2_/Sepolite-P nanoparticles by the precipitation method are as follows. The Sep was dispersed in a deionized aqueous solution following the method described above. A certain amount of potassium permanganate solution, L-ascorbic acid solution, and 5M NaOH solution were prepared. Both solutions are added dropwise to the sepiolite dispersion at the same time. And the pH alkalinity was adjusted to ensure that the solution was stable (pH = 10). It was stirred for 12 h at 105 °C under reflux. After the reaction, the mixture was centrifuged and washed three times with absolute ethanol and water, dried overnight at 105 °C, and finally ground into powder, and the powder was roasted at 350 °C for 5 h to obtain MnO_2_/Sepolite by the precipitation method. The sample was labeled as MnO_2_/Sepolite-P.

#### 3.2.3. Synthesis of MnO_x_/Sepolite-H Nanoparticles by Hydrothermal Method

The synthesis of MnO_x_/Sepiolite-H nanoparticles by the hydrothermal method was similar to the initial process of the impregnation method. The potassium permanganate solution was slowly added dropwise to the sepiolite fiber aqueous suspension at a rate of 2 s/drop and stirred at room temperature. Subsequently, the L-ascorbic acid was slowly added dropwise to the above mixture at a speed of 2 s/drop and stirred for 1 h at room temperature. And then, the resulting solution was transferred to a Teflon-lined autoclave (100 mL in capacity) and maintained at 140 °C for 12 h. After cooling, the resulting precipitate was isolated by filtration and washed with deionized water and ethanol three times. The sample was then dried at 105 °C overnight. The powder was roasted at 350 °C for 5 h to obtain MnO_2_/Sepolite by the hydrothermal method. The sample was labeled as MnO_x_/Sep-H.

### 3.3. Catalysts Characterization

X-ray diffraction (XRD) patterns of three samples were recorded on an X-ray diffractometer (Ultima IV, CuKα radiation 40 kV/40 mA, the step of 0.1 °/s, 2θ = 5–90°). Fourier-transform infrared spectra (FTIR) of samples were collected on a Nicolet 6700 spectrometer (400 to 4000 cm^−1^, 1.0 cm^−1^ interval). The N_2_ adsorption–desorption isotherms, surface area and pore size distribution of as-prepared samples were characterized on a Quantachrome NOVA-2200e based on Brunauer–Emmett–Teller (BET) model [50,51]. Transmission electron microscope (TEM) and elemental mapping micrographs were obtained through a transmission electron microscope (Talos F200X (FEI)). The samples were dispersed in alcohol with ultrasonic treatment for 20 min, and then uniformly dispersed in copper mesh for drying. X-ray photoelectron spectroscopy (XPS) measurements of samples was tested on instrument (Thermo Escalab 250Xi, Thermo Scientific, Waltham, MA, USA) equipped with an Al Kα X-ray source to determine the elemental composition and chemical states of the elements. The XPS spectra were deconvoluted by using a commercially available data-fitting program (XPSPEAK-41 software 2.0, Informer Technologies, Inc., Los Angeles, CA, USA) after a Shirley background subtraction procedure, and the binding energies were calibrated by referencing the C 1s at 284.8 eV.

H_2_ temperature-programmed reduction (H_2_-TPR) and CO_2_ temperature-programmed desorption (CO_2_-TPD) profiles were obtained from an Auto Chem II 2920 instrument equipped with a thermal conductivity detector (TCD). For H_2_-TPR, 0.1 g catalysts were heated to 200 °C with N_2_ gas (30 mL/min, 10 °C/min) for 30 min. After it cooled to room temperature, the catalyst was heated in pure H_2_ from 20 °C to 900 °C at a heating rate of 10 °C/min. For CO_2_-TPD, 0.1 g catalysts were heated to 200 °C with N_2_ gas (30 mL/min, 10 °C/min) for 30 min. After it cooled to room temperature, the sample was cleaned with pure CO_2_ or NH_3_ (50 mL/min) at room temperature, and then cleaned with 50 mL/min N_2_ for 30 min to remove the physically adsorbed CO_2_ or NH_3_. Once the baseline was stable, the temperature rose from room temperature to 900 °C at a rate of 10 °C/min.

The O_2_-TPD analysis of samples was measured on Auto Chem II 2920, 0.1 g catalysts were placed in a u-shaped tube and heated from room temperature to 200 °C and pretreated with He (50 mL/min) for 1 h to remove the surface-adsorbed water and then cooled to room temperature. The samples were then cleaned with 50 mL/min O_2_ at room temperature for 1 h and then cleaned with 50 mL/min He for 30 min to remove the physically adsorbed oxygen. Once the baseline was stable, the temperature rose from room temperature to 900 °C with a heating rate of 10 °C/min.

### 3.4. Catalyst Evaluation

**Scheme 1:**
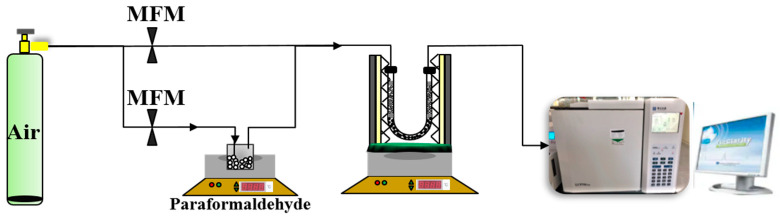
The test route of catalytic activities [43].

The catalytic activities of samples for HCHO oxidation were performed in a continuous-flow fixed-bed reactor [43]. 0.5 g catalysts (40–60 mesh) were loaded in a quartz reactor (6 mm). Gaseous HCHO was generated by purging paraformaldehyde with air. Another stream of air mixed with the gaseous HCHO. The HCHO concentration was controlled by adjusting the flow rate by a mass flow controller (GFC17A, Aalborg, New York, NY, USA.) and the temperature of incubator. The test route of the catalytic activities is shown in Scheme 1.

The oxidation product (CO_2_) was analyzed by gas chromatograph equipped with a hydrogen flame ionization detector (FuLi 7890B) and a thermal conductivity detector (TCD) (Porapak-Q column and 5A molecular sieve column). The HCHO conversion was calculated by the following equations:

$$\mathrm{HCHO}\;\mathrm{conversion}(\%)=\frac{{\left[{CO}_{2}\right]}_{\mathrm{out}}}{{\left[\mathrm{HCHO}\right]}_{\mathrm{in}}}\times 100$$

where [*CO*_2_]*_out_* (ppm) and [*HCHO*]*_in_* (ppm) are the concentrations of CO_2_ in the outlet gas and the concentrations of HCHO in the inlet gas, respectively.

## 4. Conclusions

The catalytic oxidation of HCHO over MnO_x_/Sep-H, MnO_x_/Sep-P, and MnO_x_/Sep-I catalysts that were produced using various techniques was examined in this work. The primary conclusions from the crystal structure, HCHO oxidation catalytic performance, and HCHO removal reaction mechanism were compiled. The MnO_x_/Sep-H catalyst can oxidize HCHO at 85 °C completely to CO_2_ with reaction conditions of 100 ppm of HCHO, reaction humidity of 50% and GHSV of 6000 mL/(g·h), but it needs to be at 95 °C and 125 °C over MnO_x_/Sep-P and MnO_x_/Sep-I with same reaction conditions. The Large specific surface area and pore size, a widely dispersed active component, a high percentage of Mn^3+^ species, and lattice oxygen concentration played an important role for excellent reactivity for HCHO oxidation. Thus, the MnO_x_/Sep-H catalyst is a promising catalytic material for HCHO removal in an enclosed environment or industrial exhaust gas, owing to its excellent catalytic, low cost, and easy synthesis method.

## Figures and Tables

**Table 1:** Physical parameters of the mesoporous materials and surface element composition of the samples.

Samples	S_BET_ (m^2^/g) ^a^	V_pore_ (cm^3^/g) ^a^	D_pore_ (nm) ^a^	Mn^3+^/Mn^4+ b^	O_L_ or O_ads_^b^	AOS Values ^c^
Sep	325.16	0.32	3.82	---	---	---
MnO_x_/Sep-H	136.13	0.32	3.82	1.67:1	10.0%	2.31
MnO_x_/Sep-I	176.77	0.33	3.42	1:1	16.4%	2.65
MnO_x_/Sep-P	229.45	0.35	3.41	1.15:1	6.7%	---

^a^ The S_BET_, V_pore_, and D_pore_ calculated by the BET method. ^b^ Surface element molar ratio calculated by peak areas of XPS. ^c^ The AOS values calculated by peak areas of XPS.

## Data Availability

Data are contained within the article.

## Supplementary Materials

The following supporting information can be downloaded at: https://www.mdpi.com/article/10.3390/molecules29122826/s1, Figure S1: The XPS patterns of C 1s of MnO_x_/Sep-H, MnO_x_/Sep-P and MnO_x_/Sep-I; Figure S2: The XPS patterns of Si 2p of MnO_x_/Sep-H, MnO_x_/Sep-P and MnO_x_/Sep-I.
