# Supplementary material for: Sepiolite-Supported Manganese Oxide as an Efficient Catalyst for Formaldehyde Oxidation: Performance and Mechanism
Source: Molecules. 2024 Jun 13;29(12):2826. doi: 10.3390/molecules29122826 (PMC11207037; doi:10.3390/molecules29122826)
Supplement: Supplementary file 1 [file molecules-29-02826-s001.zip › molecules-3046694-supplementary.pdf]

Supporting Information

Table of Contents

1. Figure S1. The XPS patterns of C 1s of MnOx/Sep-H, MnOx/Sep-P and MnOx/Sep-I..2

2. Figure S2. The XPS patterns of Si 2p of MnOx/Sep-H, MnOx/Sep-P and MnOx/Sep-I..3

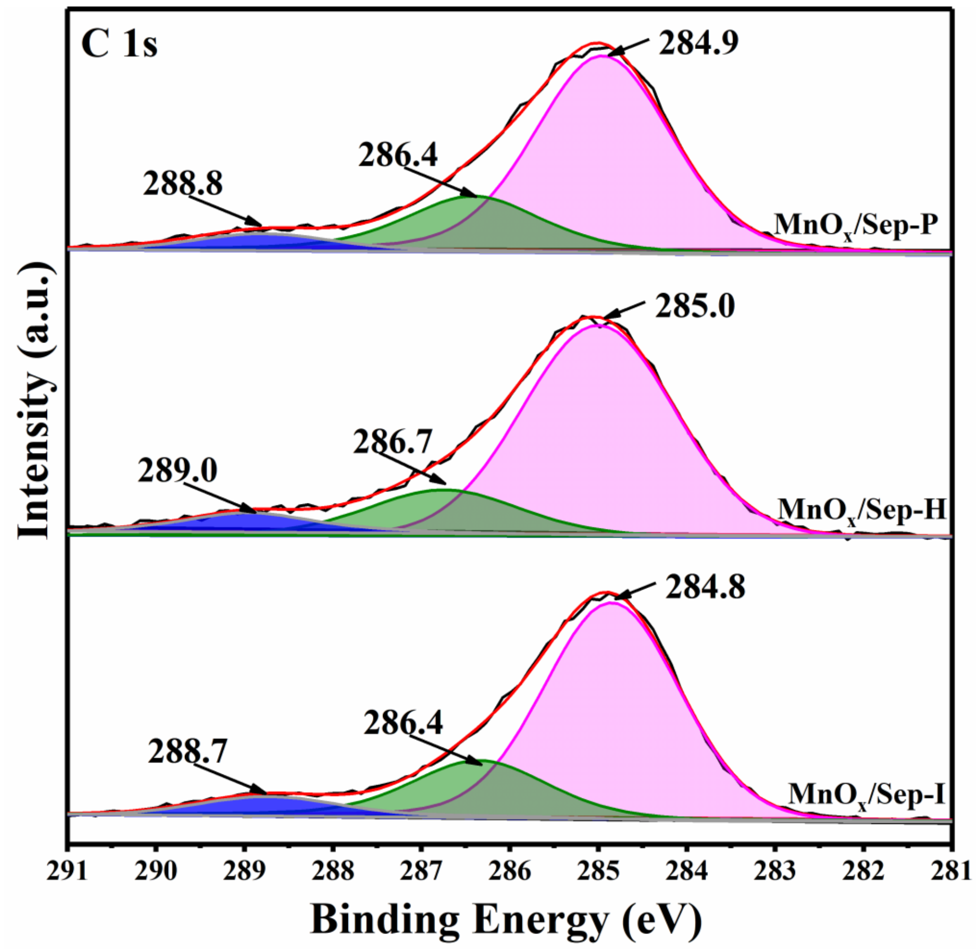

Figure S1. The XPS patterns of C 1s of MnOx/Sep-H, MnOx/Sep-P and MnOx/Sep-I.

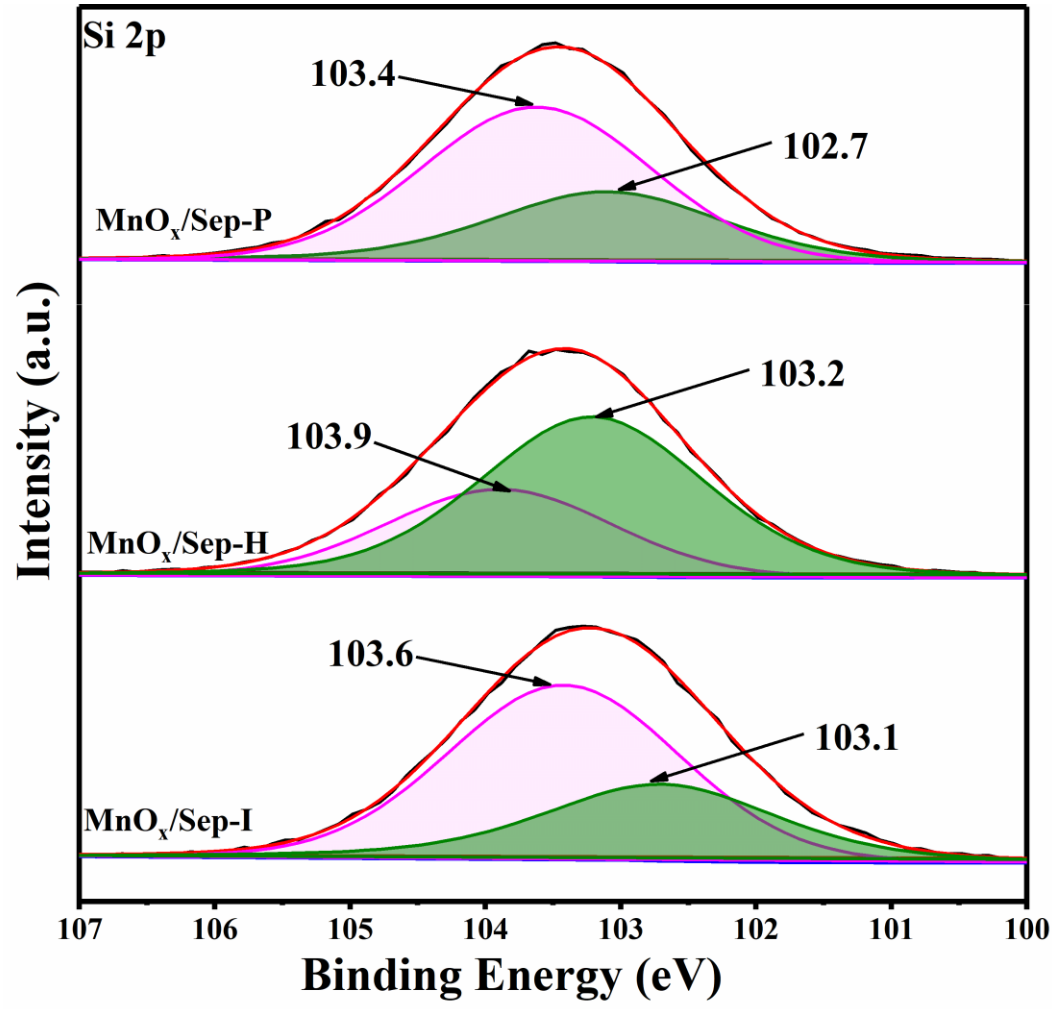

Figure S2. The XPS patterns of Si 2p of MnOx/Sep-H, MnOx/Sep-P and MnOx/Sep-I.
